# Early Life Growth Predicts Pubertal Development in South African Adolescents^1^,^2^,^3^

**DOI:** 10.3945/jn.115.222000

**Published:** 2016-02-03

**Authors:** Elizabeth A Lundeen, Shane A Norris, Reynaldo Martorell, Parminder S Suchdev, Neil K Mehta, Linda M Richter, Aryeh D Stein

**Affiliations:** 4 Nutrition and Health Sciences Program, Laney Graduate School, and; 5 Hubert Department of Global Health, Rollins School of Public Health, Emory University, Atlanta, GA; and; 6 Medical Research Council (MRC) Developmental Pathways for Health Research Unit, and; 7 Department of Science and Technology - National Research Foundation (DST-NRF) Centre of Excellence of Human Development, University of the Witwatersrand, Johannesburg, South Africa

**Keywords:** adolescent, body mass index, child growth, latent class growth analysis, puberty

## Abstract

**Background:** Given global trends toward earlier onset of puberty and the adverse psychosocial consequences of early puberty, it is important to understand the childhood predictors of pubertal timing and tempo.

**Objective:** We examined the association between early growth and the timing and tempo of puberty in adolescents in South Africa.

**Methods:** We analyzed prospectively collected data from 1060 boys and 1135 girls participating in the Birth-to-Twenty cohort in Soweto, South Africa. Height-for-age *z* scores (HAZs) and body mass index–for-age *z* scores (BMIZs) were calculated based on height (centimeters) and body mass index (kilograms per meter squared) at ages 5 y and 8 y. The development of genitals, breasts, and pubic hair was recorded annually from 9 to 16 y of age with the use of the Tanner sexual maturation scale (SMS). We used latent class growth analysis to identify pubertal trajectory classes and also characterized children as fast or slow developers based on the SMS score at 12 y of age. We used multinomial logistic regression to estimate associations of HAZ and BMIZ at ages 5 and 8 y with pubertal development.

**Results:** We identified 3 classes for pubic hair development (for both girls and boys) and 4 classes for breast (for girls) and genital (for boys) development. In girls, both HAZ and BMIZ at age 5 y were positively associated with pubic hair development [relative risk ratio (RRR): 1.57, *P* < 0.001 and RRR: 1.51, *P* < 0.01, respectively], as was BMI at age 8 y (RRR: 2.06, *P* = 0.03); similar findings were observed for breast development. In boys, HAZ and BMIZ at age 5 y were positively associated with pubic hair development (RRR: 1.78, *P* < 0.001 and RRR: 1.43, *P* < 0.01, respectively); HAZ at age 5 y was associated with development of genitals (RRR: 2.19, *P* < 0.01).

**Conclusion:** In boys and girls, both height and body mass index in early childhood predicted the trajectory of pubertal development. This may provide a tool to identify children at risk of early pubertal onset.

## Introduction

Puberty involves physical, social, and emotional changes that can have profound social implications (1–3). In girls, early puberty is associated with a higher risk of depression, eating disorders, substance abuse, poor academic achievement, and risky sexual behaviors (2). In boys, although there has been less research, a 2012 review suggests that early puberty is also a strong risk factor for detrimental psychosocial outcomes (4).

Earlier onset of puberty is occurring globally. In the United States and Western European countries, there was a marked decline in the age of menarche from the mid-19th century to the mid-20th century (5). The rate of decline in age at menarche in these countries has slowed over the past few decades. In low- and middle-income countries, which have experienced socioeconomic developments more recently than the United States and Europe have, there continues to be a strong secular trend in the reduction of menarcheal age (5, 6). Studies have also shown a secular trend toward earlier development of breasts and pubic hair (7–11). There is some research to suggest earlier genital and pubic hair development in boys, although there are far fewer data for boys, and the evidence has generally been insufficient to determine secular trends (10–13).

Trends across many populations show that the shift in age at pubertal onset has been concurrent with increases in height, suggesting that improved nutrition and socioeconomic conditions have contributed to both trends (14). In addition, studies within several populations have shown an inverse association between height and age of pubertal onset (15–18). Furthermore, the secular trends in pubertal development have been accompanied by an increase in BMI in children (19, 20), and studies on the role of overweight and obesity in pubertal development have garnered much attention (8, 21–23). There is evidence of an association between higher BMI in childhood or adolescence and earlier onset of puberty in girls (8, 15, 18, 22, 24–28). This relation has been far less studied in boys, but 2 studies concluded that higher BMI may instead be associated with delayed puberty in boys (25, 26).

Given the focus on improving early-childhood nutrition and the growth in low- and middle-income countries, it is important to better understand the relation between child growth and pubertal development in boys and girls. In addition, most studies have focused on the relation between childhood anthropometric measures and pubertal timing (often ascertained retrospectively as the age at which menses commenced), rather than examining the overall trajectory of pubertal development. Because both pubertal timing (age of onset) and tempo (rate of progression) may be influential in predicting later health and psychosocial outcomes, it is critical to use longitudinal data to better characterize these and understand their relation with child growth. Few studies, to our knowledge, have the longitudinal pubertal data needed to provide evidence on the relation between the rate of pubertal progression and later health and psychosocial outcomes. Lastly, studies on child growth and puberty mainly have focused on Caucasian children in high-income countries, with limited evidence from other populations.

We addressed these gaps in the literature by using data on the development of secondary sexual characteristics from 9 to 16 y of age to describe the longitudinal progression of puberty and its relation to childhood height and BMI (and specifically changes in height and BMI between ages 5 and 8 y), in prospectively studied adolescents in South Africa.

## Methods

### 

We analyzed data from the Birth-to-Twenty study, a birth cohort initiated in 1990 in Soweto, the largest urban township in South Africa, adjacent to Johannesburg. Detailed information on this cohort has been published elsewhere (29). The study was designed to track the growth, health, and well-being of children. The cohort enrolled 3273 children who were born during a 6-wk period in early 1990. Participants were predominantly Black, and from families with a low socioeconomic status. Ethical approval was obtained from the University of the Witwatersrand Committee for Research on Human Subjects (approval identification no. M010556), and participants or their caregivers provided written informed consent throughout the study.

Weight at birth, and weight and length/height at subsequent annual rounds were measured with the use of standard procedures (30). BMI was calculated as weight (kilograms) divided by height squared (meters squared). Height and BMI measures were converted to height-for-age *z* scores (HAZs)^8^ and BMI-for-age *z* scores (BMIZs) with the use of the WHO standards (31, 32). At birth or soon thereafter, data on demographic and socioeconomic variables, including the child’s ethnicity and maternal monthly income, were collected. Income was categorized into quintiles.

At 9–16 y of age, pubertal development was assessed with the Tanner sexual maturation scale (SMS), a commonly used standard for evaluating pubertal stage in both clinical and research settings (33). The SMS consists of drawings of progressive stages in the development of secondary sexual characteristics—breasts in girls, genitals in boys, and pubic hair in both sexs (34, 35). The drawings are accompanied by written descriptions of each stage. The stages range from 1 (prepubertal) to 5 (postpubertal), with stage 2 considered to be the onset of puberty. From 9 to 11 y of age, a trained health care provider administered the SMS in a subset of Birth-to-Twenty participants. In a comparable group of South African adolescents, self-assessment was validated against expert assessment, both with the use of the SMS, and a substantial degree of concordance was found between the 2 ratings (36). Based on these findings, pubertal self-rating with the use of SMS was carried out at 12–16 y of age in the entire cohort.

Participants were included in the present analysis if they had at least one SMS measurement, which resulted in an analytical sample of 2195 participants. Missing data were accounted for with the use of the Full Information Maximum Likelihood technique (37).

#### Data management.

We used latent class growth analysis (LCGA) in order to group participants into distinct classes based on common developmental trajectories for the SMS indicators of pubertal stage (**Supplemental Figure 1**). LCGA is an extension of latent growth curve modeling, which estimates a latent intercept (initial level of the outcome variable) and latent slope (rate of outcome change over time) for the entire population. However, instead of assuming that one growth trajectory adequately describes the entire population, LCGA identifies multiple growth trajectories and groups observations into distinct classes based on similarities in their growth patterns. We conducted separate analyses for girls (breast and pubic hair development), and for boys (genital and pubic hair development). We determined the optimal model in terms of the number of classes based on model fit statistics, with a focus on minimizing the value for the Bayesian Information Criteria, as well as parsimony of the model and identifying classes that had distinct differences in their developmental trajectories (37).

We used ANOVA to assess the significance of differences in HAZ and BMIZ at ages 5 and 8 y between classes. We used multinomial logistic regression to determine whether HAZ and BMIZ at ages 5 and 8 y were associated with class membership. To address the strong correlation between repeated measures of HAZ and BMIZ in the same individuals, we computed sex-specific conditional measures of HAZ and BMIZ at age 8 y, as described elsewhere (38). These conditional measures represent the change in each of these measures that is not explained by the prior measure. A positive value represents a child who is growing faster than expected and a negative value characterizes a child growing slower than expected from their measure at age 5 y and the mean pattern in the sample. The results of the multinomial regression analyses are presented as relative risk ratios (RRRs), which are interpretable as ORs.

To facilitate more direct comparisons with the existing literature, we created an indicator to represent the timing of pubertal development by comparing the children’s level of development in early adolescence to that of their peers. SMS scores at age 12 y were used, because the full cohort began providing pubertal data at this wave. Children who were at the median score at 12 y of age for pubic hair, breast, and genital development were considered to be the reference category. Those with a lower score were classified as slower developers, and those with a higher score were considered to be more rapid developers. With these 3 categories, we used multinomial logistic regression to assess the association between HAZ and BMIZ at ages 5 and 8 y with stage of pubertal development at age 12 y. We adjusted all models for ethnicity (Black, White, Indian, and Coloured) and quintiles of income. We used Mplus 7.3 (Muthén & Muthén) to perform the LGCA and to handle the missing data, and STATA 13.0 for all other analyses. *P* < 0.05 was considered to be statistically significant.

## Results

The study sample was 52% female (**Table 1**). The large majority (81%) of participants were Black, with other represented groups including White (4%), Indian (2%), and Coloured or mixed-ancestral (13%) children. Those who were not included in the analysis were similar with respect to ethnicity, maternal income, and height and BMI at ages 5 and 8 y. At ages 5 and 8 y, both girls and boys had a mean HAZ of −0.7 SD. At age 5 y, girls had a mean BMIZ of 0.1 SD, and boys had a mean BMIZ of 0.2. At age 8 y, both girls and boys had a mean BMIZ of −0.1 SD.

**TABLE 1:** Selected characteristics of the study population by sex, Birth-to-Twenty study^1^

	Girls (*n* = 1135)	Boys (*n* = 1060)
Ethnicity		
Black	81.3	81.2
White	3.4	4.0
Coloured or mixed-ancestral	13.0	12.5
Indian	2.4	2.4
Maternal monthly income quintiles^2^		
1 (0–2400 rands)	23.9	21.9
2 (2401–7800 rands)	28.6	32.4
3 (7801–10,800 rands)	16.2	13.4
4 (10,801–19,200 rands)	21.0	20.2
5 (19,201–48,000 rands)	10.3	12.2
Height, cm		
5 y	106.9 ± 4.7	107.6 ± 4.5
8 y	123.9 ± 6.0	124.7 ± 6.0
Height-for-age *z* score		
5 y	−0.7 ± 0.9	−0.7 ± 0.9
8 y	−0.7 ± 1.0	−0.7 ± 1.0
BMI, kg/m^2^		
5 y	15.6 ± 1.5	15.7 ± 1.4
8 y	15.8 ± 2.0	15.8 ± 1.7
BMI *z* score		
5 y	0.1 ± 0.9	0.2 ± 1.0
8 y	−0.1 ± 1.0	−0.1 ± 1.0

1 Values are means ± SDs or percentages.

2 A total of 623 girls and 575 boys had information on maternal monthly income. In the analyses that used this variable, a sixth category for missing information was included to preserve the sample size. In 1990, the South African rand–to–US dollar conversion rate was 0.39.

At age 9 y, when collection of pubertal data began, the large majority of children were at stage 1 for pubic hair, breasts, and genitals (**Table 2**, **Supplemental Table 1**). Over one-half were still at stage 1 at 10 y of age; by age 11 y, the majority had reached stage 2 or greater. At ages 12 and 13 y, girls had reached a higher level of pubertal development than boys. By age 16 y, approximately one-half of the adolescents were at stage 4, and around one-fourth had reached stage 5.

**TABLE 2:** Mean age at Tanner Pubertal Development Stages by sex and ethnicity, Birth-to-Twenty study^1^

	Black		White		Coloured/mixed-ancestral		Indian	
	*n*	Value	*n*	Value	*n*	Value	*n*	Value
Girls								
Age at pubic hair development stages, y								
Tanner 2	144	12.2 ± 1.5	15	12.1 ± 0.9	6	14.4 ± 1.4	1	13.5^2^
Tanner 3	318	14.0 ± 1.3	16	13.3 ± 1.2	20	15.0 ± 1.5	4	15.1 ± 1.6
Tanner 4	580	15.1 ± 1.4	23	14.6 ± 1.1	60	15.4 ± 1.4	9	16.1 ± 1.0
Tanner 5	307	16.3 ± 1.4 (16.4)	13	15.6 ± 1.1 (15.5)	55	16.3 ± 1.4 (16.3)	7	17.2 ± 1.1 (16.6)
Age at breast development stages, y								
Tanner 2	123	11.9 ± 1.2	18	11.8 ± 1.2	1	12.4 ± ^2^	2	13.6 ± 0.03
Tanner 3	308	13.8 ± 1.4	17	12.9 ± 1.5	19	14.5 ± 1.5	6	15.1 ± 1.5
Tanner 4	559	14.9 ± 1.4	22	15.0 ± 1.7	61	15.3 ± 1.6	7	16.1 ± 0.6
Tanner 5	383	16.0 ± 1.5 (15.9)	11	16.5 ± 1.2 (16.4)	48	16.4 ± 1.3 (16.1)	4	17.8 ± 1.1 (17.9)
Boys								
Age at pubic hair development stages, y								
Tanner 2	203	12.4 ± 1.5	30	11.2 ± 0.8	8	14.2 ± 1.2	0	—^2^
Tanner 3	343	14.2 ± 1.2	16	13.1 ± 0.6	17	14.9 ± 0.7	0	—^2^
Tanner 4	550	15.4 ± 1.2	17	14.2 ± 0.7	63	15.9 ± 1.2	4	14.7 ± 1.1
Tanner 5	322	16.7 ± 1.2 (16.8)	20	16.4 ± 1.0 (16.2)	44	16.9 ± 1.2 (17.0)	11	16.8 ± 1.5 (16.7)
Age at genital development stages, y								
Tanner 2	203	12.6 ± 1.6	20	11.4 ± 1.1	9	14.2 ± 1.7	0	—^2^
Tanner 3	401	14.2 ± 1.4	18	12.9 ± 1.2	20	14.2 ± 1.0	0	—^2^
Tanner 4	560	15.4 ± 1.3	20	14.4 ± 0.9	62	15.9 ± 1.3	8	15.9 ± 1.7
Tanner 5	275	16.6 ± 1.3 (16.7)	19	16.3 ± 0.9 (16.4)	41	16.9 ± 1.3 (17.0)	7	16.5 ± 1.1 (16.7)

1 Values are means ± SDs; (medians) also are shown for Tanner stage 5 because of right-censored data—not all participants had reached Tanner stage 5 by the end of the follow-up. Of those who ever reported being at the previous Tanner stage, the age is that at which the transition to the specified Tanner stage was documented. The distribution of participants across Tanner stages by age at data collection is presented in Supplemental Table 1.

2 Insufficient sample size. With a sample size of 0 or 1, a mean ± SD cannot be calculated.

**FIGURE 1:**
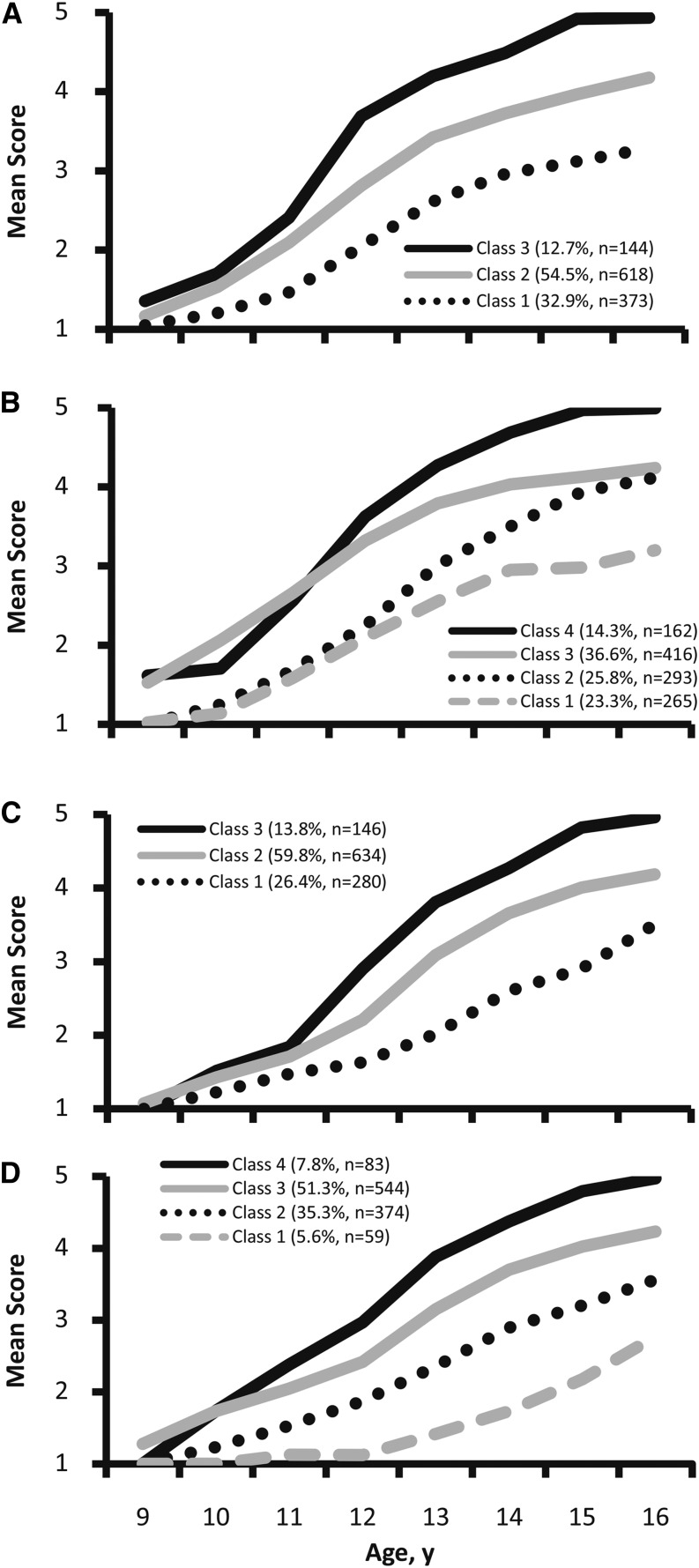
Mean Tanner scores for female (*n* = 1135) and male (*n* = 1060) pubertal development from 9 to 16 y of age by growth trajectory class and age, Birth-to-Twenty study. Pubic hair, girls (A); breasts, girls (B); pubic hair, boys (C); and genitals, boys (D).

In girls, LCGA identified 3 trajectory classes for the development of pubic hair (**Table 3**, **Figure 1A**) and 4 classes for breast development (Figure 1B). In boys, 3 trajectory classes were identified for the development of pubic hair (Figure 1C) and 4 classes for genital development (Figure 1D). In the development of pubic hair in girls, for example, class 3, with the largest intercept and slope, represents girls who started at a slightly higher level of pubertal maturation at 9 y of age, had a faster tempo of progression through the Tanner stages, and were at a higher stage of development at age 16 y (Figure 1A). Girls in classes 1 and 2 had a slower pace of pubic hair development and had not reached postpubertal status by 16 y of age. In all cases, we consider class 1 (children who are at the lowest Tanner stage at age 9 y and progress slowest over time) to be the reference category.

**TABLE 3:** Percentage within class and intercept and slope for the pubertal growth trajectory classes (derived with the use of Tanner scores from 9 to 16 y of age) by sex, Birth-to-Twenty study^1^

	Girls (*n* = 1135)			Boys (*n* = 1060)		
Outcome	%	Intercept	Slope	%	Intercept	Slope
Pubic hair						
Class 1	32.9	0.00	0.97	26.4	−0.63	1.20
Class 2	54.5	0.79	1.32	59.8	0.00	1.61
Class 3	12.7	1.16	1.98	13.8	−0.72	2.38
Breast (girls)						
Class 1	23.3	0.00	0.94			
Class 2	25.8	−0.92	1.56			
Class 3	36.6	2.58	1.17			
Class 4	14.3	0.84	2.24			
Genital (boys)						
Class 1				5.6	−3.41	1.23
Class 2				35.3	−0.54	1.15
Class 3				51.3	0.33	1.38
Class 4				7.8	0.00	1.94

1 Higher class numbers represent children who were at a more advanced stage of pubertal development by age 16 y, either because they started out at a more advanced stage at age 9 y, or because they progressed through pubertal development more quickly (or both).

Mean height and BMI at ages 5 and 8 y differed across growth trajectory classes (**Table 4**). Differences across classes in mean height and BMI were statistically significant (*P* < 0.05), with the exception of height at age 8 y and female pubic hair development, and BMI at age 5 y and male genital development.

**TABLE 4:** Height and BMI at 5 and 8 y of age, by Pubertal Growth Trajectory Class and sex, Birth-to-Twenty study^1^

	Height, cm				BMI, kg/m^2^			
	Year 5		Year 8		Year 5		Year 8	
	Value	*P*^2^	Value	*P*^2^	Value	*P*^2^	Value	*P*^2^
Girls^3^								
Pubic hair		<0.001		0.1090		0.0016		<0.001^4^
Class 1	106.0 ± 4.8		123.2 ± 5.8		15.4 ± 1.5		15.5 ± 1.7	
Class 2	107.2 ± 4.5		124.1 ± 6.0		15.6 ± 1.5		16.0 ± 2.2	
Class 3	108.0 ± 4.9		124.8 ± 6.3		16.0 ± 1.4		16.5 ± 1.9	
Breast		<0.001		<0.001		<0.001^4^		<0.001^4^
Class 1	106.0 ± 4.7		123.2 ± 5.9		15.3 ± 1.6		15.2 ± 1.6	
Class 2	106.1 ± 4.5		122.4 ± 5.7		15.3 ± 1.2		15.4 ± 1.3	
Class 3	107.5 ± 4.7		124.9 ± 5.9		15.7 ± 1.6		16.2 ± 2.4	
Class 4	108.3 ± 4.4		125.5 ± 5.9		16.1 ± 1.5		17.0 ± 2.5	
Boys^5^								
Pubic hair		<0.001		0.0023		0.0194		0.0100^4^
Class 1	106.6 ± 4.3		123.7 ± 5.8		15.5 ± 1.3		15.6 ± 1.5	
Class 2	107.8 ± 4.5		124.8 ± 6.1		15.7 ± 1.5		15.8 ± 1.8	
Class 3	109.0 ± 4.6		126.5 ± 5.7		16.0 ± 1.4		16.2 ± 1.4	
Genital		<0.001		<0.001		0.11^4^		0.0094^4^
Class 1	106.0 ± 3.5		123.2 ± 5.0		15.4 ± 1.4		15.2 ± 1.1	
Class 2	106.9 ± 4.7		123.8 ± 6.2		15.7 ± 1.5		16.0 ± 1.9	
Class 3	108.1 ± 4.4		125.2 ± 5.8		15.6 ± 1.3		15.7 ± 1.6	
Class 4	109.2 ± 4.3		127.4 ± 6.0		16.0 ± 1.2		16.2 ± 1.4	

1 Values are means ± SDs. Higher class numbers represent children who were at a more advanced stage of pubertal development by age 16 y, either because they started out at a more advanced stage at age 9 y, or because they progressed through pubertal development more quickly (or both).

2 ANOVA test to determine whether there was a significant difference in mean height and BMI between groups.

3 Sample size for pubic hair in girls was *n* = 808 at age 5 y and *n* = 615 at age 8 y (for both height and BMI), and for breasts in girls it was *n* = 809 at age 5 y and *n* = 615 at age 8 y (for both height and BMI).

4 Simulated ANOVA test used because the equal variances assumption was not met.

5 Sample size for pubic hair in boys was *n* = 734 at age 5 y and *n* = 603 at age 8 y (for both height and BMI), and for genitals in boys it was *n* = 734 at age 5 y and *n* = 603 at age 8 y (for both height and BMI).

In girls, a 1 SD increase in HAZ at age 5 y was associated with a 57% increase in the risk of being in class 3 compared with class 1 for pubic hair development, and a 34% increase in the risk of being in class 2 compared with class 1 (**Table 5**, **Supplemental Table 2**). For breast development, a HAZ at age 5 y was associated with a significantly increased RRR for class 4 compared with class 1, and class 3 compared with class 1. In girls, a conditional HAZ at age 8 y was not significant in predicting group membership for either pubic hair or breast development. When trajectory classes for pubic hair development were regressed on BMIZ at age 5 y, there was a significantly increased RRR for class 3 compared with class 1; for conditional BMIZ at age 8 y, significant RRRs were found for class 3 compared with class 1, and class 2 compared with class 1. For breast development, BMIZ at age 5 y and conditional BMIZ at age 8 y both were associated with a significantly increased likelihood of being in class 3 or 4.

**TABLE 5:** Regression to predict Pubertal Development Trajectory Class based on HAZ and BMIZ at ages 5 and 8 y by sex, Birth-to-Twenty study^1^

	Age 5 y				Age 8 y			
	Girls (*n* = 808)		Boys (*n* = 734)		Girls (*n* = 526)		Boys (*n* = 500)	
	Adjusted RRR^2^	95% CI	Adjusted RRR^2^	95% CI	Adjusted RRR^3^	95% CI	Adjusted RRR^3^	95% CI
HAZ								
Pubic hair^4^								
Class 3 vs. class 1	1.57	(1.22, 2.03)	1.78	(1.35, 2.34)	1.03	(0.57, 1.86)	1.57	(0.80, 3.07)
Class 2 vs. class 1	1.34	(1.13, 1.59)	1.32	(1.10, 1.60)	0.93	(0.65, 1.34)	1.07	(0.68, 1.67)
Breast/genital^5^								
Class 4 vs. class 1	1.78	(1.37, 2.33)	2.19	(1.41, 3.40)	0.95	(0.52, 1.74)	1.99	(0.62, 6.37)
Class 3 vs. class 1	1.39	(1.13, 1.72)	1.68	(1.19, 2.38)	0.83	(0.52, 1.32)	1.24	(0.51, 3.05)
Class 2 vs. class 1	0.97	(0.77, 1.22)	1.28	(0.90, 1.81)	0.74	(0.46, 1.20)	0.68	(0.28, 1.66)
BMIZ								
Pubic hair^4^								
Class 3 vs. class 1	1.51	(1.17, 1.96)	1.43	(1.11, 1.85)	2.06	(1.10, 3.87)	1.00	(0.56, 1.79)
Class 2 vs. class 1	1.09	(0.91, 1.30)	1.15	(0.96, 1.37)	1.58	(1.06, 2.34)	0.70	(0.46, 1.05)
Breast/genital^5^								
Class 4 vs. class 1	1.65	(1.25, 2.17)	1.49	(0.99, 2.26)	3.35	(1.74, 6.46)	1.40	(0.50, 3.94)
Class 3 vs. class 1	1.25	(1.01, 1.55)	1.19	(0.85, 1.66)	2.56	(1.52, 4.33)	1.56	(0.68, 3.56)
Class 2 vs. class 1	1.00	(0.79, 1.27)	1.31	(0.93, 1.85)	1.75	(1.03, 2.97)	1.48	(0.64, 3.41)

1 Higher class numbers represent children who were at a more advanced stage of pubertal development by age 16 y, either because they started out at a more advanced stage at age 9 y, or because they progressed through pubertal development more quickly (or both). BMIZ, BMI-for-age *z* score; HAZ, height-for-age *z* score; RRR, relative risk ratio.

2 Adjusted for ethnicity and quintiles of maternal monthly income. BMIZ at age 5 y is adjusted for HAZ at age 5 y.

3 Adjusted for HAZ and BMIZ at age 5 y, ethnicity, and quintiles of maternal monthly income. HAZ at age 8 y is included in the model as the residual produced when HAZ at age 8 y is regressed on HAZ at age 5 y. BMIZ at age 8 y is included in the model as the residual produced when BMIZ at age 8 y is regressed on BMIZ at age 5 y.

4 Multinomial logistic regression with the outcome being the growth trajectory groups for pubic hair development.

5 Multinomial logistic regression with the outcome being the growth trajectory groups for breast development in girls and genital development in boys.

In boys, we found significantly increased adjusted RRRs for class 3 compared with class 1, and class 2 compared with class 1 for pubic hair development, and class 4 compared with class 1, and class 3 compared with class 1 for genital development (Table 5). A greater BMIZ at age 5 y, but not a conditional BMIZ at age 8 y, was associated with being in class 3 compared with class 1 for pubic hair development.

In girls, the median SMS score for both pubic hair and breast development at age 12 y was 3. For pubic hair development, 38% were at stage 3, with 19% classified as more advanced and 43% as progressing more slowly (data not shown). For breast development, 40% were at stage 3, with 23% classified as more advanced and 37% as progressing more slowly. A greater HAZ at age 5 y and a greater conditional HAZ at age 8 y were associated with an increased likelihood of being more advanced with respect to pubic hair development, and with a lower risk of being slow to progress for both pubic hair and breast development (**Table 6**, **Supplemental Table 3**). A greater BMIZ at age 5 y increased the risk of being more advanced for pubic hair development at age 12 y.

**TABLE 6:** Regression to predict pubertal development group at 12 y of age based on HAZ and BMIZ at ages 5 and 8 y by sex, Birth-to-Twenty study^1^

	Age 5 y				Age 8 y			
	Girls (*n* = 808)		Boys (*n* = 734)		Girls (*n* = 526)		Boys (*n* = 500)	
	Adjusted RRR^2^	95% CI	Adjusted RRR^2^	95% CI	Adjusted RRR^3^	95% CI	Adjusted RRR^3^	95% CI
HAZ								
Pubic hair^4^								
More advanced	1.40	(1.09, 1.81)	1.24	(1.00, 1.55)	2.33	(1.25, 4.35)	0.91	(0.55, 1.52)
Less advanced	0.72	(0.59, 0.89)	0.78	(0.61, 1.00)	1.06	(0.68, 1.66)	1.02	(0.56, 1.87)
Missing	0.85	(0.68, 1.06)	1.02	(0.82, 1.28)	1.13	(0.69, 1.87)	0.64	(0.37, 1.11)
Breast/genital^5^								
More advanced	1.12	(0.88, 1.43)	1.18	(0.95, 1.47)	1.10	(0.63, 1.92)	0.82	(0.49, 1.36)
Less advanced	0.65	(0.52, 0.80)	0.77	(0.60, 0.98)	0.96	(0.61, 1.51)	1.05	(0.57, 1.92)
Missing	0.80	(0.64, 0.99)	1.05	(0.84, 1.31)	1.01	(0.63, 1.64)	0.63	(0.36, 1.10)
BMIZ								
Pubic hair^4^								
More advanced	1.32	(1.02, 1.70)	1.01	(0.82, 1.24)	1.25	(0.68, 2.28)	1.43	(0.88, 2.30)
Less advanced	0.96	(0.78, 1.19)	0.80	(0.63, 1.01)	0.73	(0.46, 1.17)	1.68	(1.00, 2.84)
Missing	1.11	(0.88, 1.39)	0.96	(0.77, 1.18)	0.85	(0.50, 1.44)	1.07	(0.63, 1.82)
Breast/genital^5^								
More advanced	1.16	(0.92, 1.48)	0.92	(0.75, 1.13)	1.68	(0.97, 2.91)	0.95	(0.59, 1.53)
Less advanced	0.86	(0.69, 1.07)	0.93	(0.74, 1.18)	0.62	(0.38, 1.01)	0.85	(0.51, 1.42)
Missing	1.06	(0.85, 1.32)	0.95	(0.77, 1.18)	0.88	(0.52, 1.48)	0.79	(0.47, 1.34)

1 BMIZ, BMI-for-age *z* score; HAZ, height-for-age *z* score; RRR, relative risk ratio.

2 Adjusted for ethnicity and quintiles of maternal monthly income. BMIZ at age 5 y is adjusted for HAZ at age 5 y.

3 Adjusted for HAZ and BMIZ at age 5 y, ethnicity, and quintiles of maternal monthly income. HAZ at age 8 y is included in the model as the residual produced when HAZ at age 8 y is regressed on HAZ at age 5 y. BMIZ at age 8 y is included in the model as the residual produced when BMIZ at age 8 y is regressed on BMIZ at age 5 y.

4 Multinomial logistic regression with the outcome being amount of pubic hair development at age 12 y: more advanced, medium (ref), less advanced, or missing.

5 Multinomial logistic regression with the outcome being amount of breast development in girls and genital development in boys at age 12 y: more advanced, medium (ref), less advanced, or missing.

In boys, the median SMS score for both pubic hair and genital development at age 12 y was 2. For pubic hair development, 44% were at stage 2, with 33% classified as more advanced and 23% as less advanced. For genital development, 44% were at stage 2, with 34% classified as more advanced and 22% as less advanced. For boys, only one association was significant, with a greater HAZ at 5 y of age associated with a reduced risk of being less advanced for genital development at age 12 y (Table 6).

## Discussion

We studied pubertal development longitudinally in a large contemporary sample of South African boys and girls. We found that, in girls, greater HAZ at age 5 y and BMIZ at age 5 y were associated with a faster tempo for pubic hair and breast development. Furthermore, in girls (but not in boys), a greater-than-average increase in BMIZ between ages 5 and 8 y was associated with earlier initiation and faster progression through puberty. In boys, a greater HAZ at age 5 y was associated with a faster tempo for pubic hair and genital development, and a greater BMIZ at age 5 y was associated with a faster tempo for pubic hair development.

These findings indicate that linear growth and BMI in early childhood are both positively associated with the pubertal development trajectory in boys and girls. For linear growth, the association was established by age 5 y, with no added explanatory power derived from the change in HAZ between ages 5 and 8 y. For BMIZ, however, greater-than-average increases between ages 5 and 8 y were predictive of pubertal transitions in girls. The associations found between BMIZ and pubertal timing and tempo were independent of HAZ.

Our results are consistent with other studies on the relation between child growth and puberty in high-income countries, which have found an inverse relation between childhood height and age of puberty onset in girls (15, 18). Several cross-sectional studies have found that a higher BMI is associated with earlier puberty in girls, as defined by breast development (24, 25), pubic hair (15, 24), and age at menarche (18, 24, 26). Our study adds to this literature by including data from boys, and considering pubertal development as a process (considering timing and tempo) with the use of prospectively collected serial data. Our study also makes an important contribution to the literature on childhood BMI and pubertal progression by exploring this association in a population that has a much lower prevalence of childhood overweight and obesity than that which is seen in higher-income countries. In our sample, the combined prevalence of overweight and obesity at 4–8 y of age was 19% in boys and 15% in girls (39).

Longitudinal studies have helped clarify the direction of these associations. One study followed White girls from 5 to 9 y of age, and found that girls with greater body fat at ages 5 and 7 y were more likely to have earlier pubertal development at age 9 y, assessed by breast development, estradiol concentrations, and the Pubertal Development Scale (27). Another study that enrolled girls at 6–8 y of age found that girls with a greater BMI reached breast stage 2 at a younger age (8). Lee et al. (28) found that BMI measured as early as age 36 mo is associated with breast development. These relations have been far less studied in boys, but 2 studies have found that a higher BMI instead may be associated with delayed puberty in boys (25, 26). Our data add to these findings by assessing the independent contributions of size at age 5 y and change in size between 5 and 8 y of age, and examining HAZ and BMIZ separately.

There have been hypothesized biological mechanisms that support a causal relation between child growth and pubertal development. For girls, both leptin and estrogen, hormones produced in adipose cells, are thought to play a role in the stimulation of pubertal development (40). Overweight girls have been found to have higher concentrations of estrogen, potentially expediting the pubertal process (41, 42). For both boys and girls, the timing of adrenarche, the maturation of the adrenal gland and increase in adrenal androgens that occurs in early childhood, may contribute to both the timing of the midchildhood growth spurt and other maturational events, such as puberty (40). Such a connection would suggest that a larger body size at 5 y of age is a marker for a child’s overall developmental trajectory.

### Limitations.

A limitation of this study is that the SMS ratings at 12–16 y of age were self-reported by the adolescents. The gold standard for pubertal staging is a physical examination by a trained clinician (33). However, self-assessment with the use of the SMS has been validated in many populations, including a highly comparable population of South African adolescents (36). Therefore, self-rating of pubertal stage can be considered as reasonably valid in this study. Also, although measurement error associated with self-assessment could affect conclusions drawn about the amount of pubertal development at any one cross-section within the study, the consistent use of self-rating from 12 to 16 y of age means that interpretation of trends across time would be unlikely to be biased, because measurement error is equally likely at all ages.

The associations we found between BMI and breast development in girls should be interpreted with caution, because it has been suggested that using the SMS to characterize breast development in the absence of physical examination by a clinician may result in a measurement error for overweight or obese girls (33). The SMS is most accurately used to describe female breast development when a trained clinician uses palpation to distinguish between actual breast tissue and adipose tissue. Overweight or obese girls who use self-assessment may be prone to overestimate their amount of breast development, because adipose tissue can be easily mistaken for breast tissue (33). In our sample, the combined prevalence of overweight and obesity in girls was 21% at 11–12 y of age, 25% at 13–15 y of age, and 27% at 16–18 y of age (39).

Our longitudinal data are limited by the fact that only a subset of the overall cohort provided data at 9–11 y of age. In girls, those with pubertal data at 9–11 y of age and those who only began providing data at age 12 y were similar with respect to Tanner scores at 12 y of age, as well as height and BMI at ages 5 and 8 y. Boys who provided pubertal data at 9–11 y of age were taller at age 5 y, had a higher BMI at age 8 y, and reported more pubic hair development at age 12 y than those who were first staged at age 12 y. These differences in the boys could have affected the group assignments for pubic hair progression, as well as the regression analyses involving these groups.

Lastly, direct comparisons between the results of our study and previous research may be difficult, because our population consisted primarily of Black adolescents. Some research suggests that Black girls experience pubertal development earlier than White girls (7, 15, 18, 24, 28). However, the small number of White children in our sample generally showed earlier pubertal development than did the Black children. This might reflect underlying patterns of nutrition in early life.

### Strengths.

Whereas many studies have found an association between child growth variables and pubertal development, our study helps to clarify this relation by examining both boys and girls, exploring the association for height, as well as BMI, and using longitudinal data to describe this relation for both pubertal timing and tempo. The collection of height and BMI measurements at 2 time points before the onset of secondary sexual characteristics helps to establish the timing of this association. Lastly, our study explores these associations in the context of a primarily Black population in a middle-income country.

In conclusion, in both boys and girls, there was a positive association between both height and BMI in early childhood and the tempo of pubertal development. In girls, greater childhood height and BMI were associated with more advanced pubertal development at age 12 y, but there was no such association in boys. Our data suggest that efforts to improve child growth may have contributed to the secular trend of lowered age at puberty, and that efforts to reduce the prevalence of overweight and obesity, especially in girls, might result in slowing this trend. Finally, our data suggest that assessing growth between 5 and 8 y of age might be useful to identify children at risk of the early onset of puberty, in whom interventions to mitigate the psychosocial consequences might be developed.

## Footnotes

8 Abbreviations used: BMIZ, BMI-for-age *z* score; HAZ, height-for-age *z* score; LCGA, latent class growth analysis; RRR, relative risk ratio; SMS, Tanner sexual maturation scale.
